# Supplementary material for: The effect of sodium carboxymethyl starch with high degree of substitution on defecation
Source: PLoS One. 2021 Sep 3;16(9):e0257012. doi: 10.1371/journal.pone.0257012 (PMC8415588; doi:10.1371/journal.pone.0257012)
Supplement: S1 Graphical abstract — (DOC) [file pone.0257012.s001.doc]

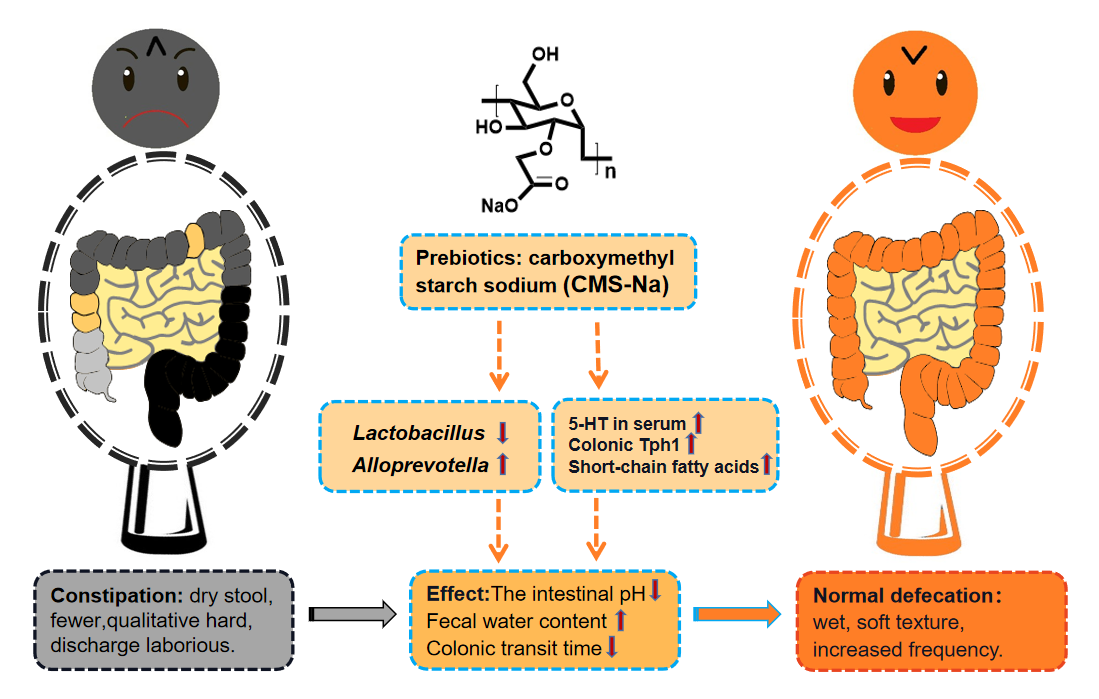


CMS-Na alleviates constipation. The mechanism may be related to regulating *Alloprevotella* and *Lactobacillus* in the colon, increasing short-chain fatty acids and promoting the synthesis of Tph1 and 5-HT.
