# Supplementary material for: The effect of sodium carboxymethyl starch with high degree of substitution on defecation
Source: PLoS One. 2021 Sep 3;16(9):e0257012. doi: 10.1371/journal.pone.0257012 (PMC8415588; doi:10.1371/journal.pone.0257012)
Supplement: Attachment — Submitted filename: Response to Reviewers.doc [file pone.0257012.s003.doc]

| **Reviewer’s Comments** | **Authors’ Response** |
| --- | --- |
| ***Journal Requirements:***  Please review your reference list to ensure that it is complete and correct. If you have cited papers that have been retracted, please include the rationale for doing so in the manuscript text, or remove these references and replace them with relevant current references. Any changes to the reference list should be mentioned in the rebuttal letter that accompanies your revised manuscript. If you need to cite a retracted article, indicate the article’s retracted status in the References list and also include a citation and full reference for the retraction notice. | Thanks you.  All of references have been reviewed in accordance with PLOS ONE guidelines. |
| ***Comment 1:***   5. Is the manuscript presented in an intelligible fashion and written in standard English?   Reviewer #2: No | Thank you for your comments.  We have checked and revised the manuscript very carefully, We believes that the revised version is significantly improved. |
| ***Comment 2:***   6. Review Comments to the Author   Reviewer #1: The quality of the figures seems low. Some fonts are too big or in some case small. The authors claimed to address the references, but it seems that the references were not implemented. | Thank you for your comments.  The quality of the figures has been improved.The size of phrases and numbers varies with the size of the graph.  the references has been deleted and improved. |
| ***Comment 3:***  6.Review Comments to the Author  Reviewer #2: The authors have adequately addressed our comments raised in a previous round of review. However, before be published, this manuscript should be carefully checked for grammatical errors. Furthermore, the clarity of all figures needs to be improved. Usually, authors should provide pictures (300DPI) in tiff format. | Thanks you.  We have corrected the grammatical and spelling mistakes throughout the article. the clarity of most of figures has been improved (300DPI). |
